# Zerumbone Inhibits *Helicobacter pylori* Urease Activity

**DOI:** 10.3390/molecules26092663

**Published:** 2021-05-01

**Authors:** Hyun Jun Woo, Ji Yeong Yang, Pyeongjae Lee, Jong-Bae Kim, Sa-Hyun Kim

**Affiliations:** 1 Department of Clinical Laboratory Science, Semyung University, Jecheon 27136, Korea; taesube@nate.com; 2 Division of Crop Foundation, National Institute of Crop Science (NICS), Rural Development Administration (RDA), Wanju 55365, Korea; yjy90@korea.kr; 3 School of Oriental Medicine and Bio Convergence Sciences, Semyung University, Jecheon 27136, Korea; pjlee1@semyung.ac.kr; 4 Department of Biomedical Laboratory Science, College of Health Sciences, Yonsei University, Wonju 26493, Korea; kimjb70@yonsei.ac.kr

**Keywords:** antimicrobial, dimerization, *H. pylori*, urease, zerumbone

## Abstract

*Helicobacter pylori* (*H. pylori*) produces urease in order to improve its settlement and growth in the human gastric epithelium. Urease inhibitors likely represent potentially powerful therapeutics for treating *H. pylori*; however, their instability and toxicity have proven problematic in human clinical trials. In this study, we investigate the ability of a natural compound extracted from *Zingiber zerumbet* Smith, zerumbone, to inhibit the urease activity of *H. pylori* by formation of urease dimers, trimers, or tetramers. As an oxygen atom possesses stronger electronegativity than the first carbon atom bonded to it, in the zerumbone structure, the neighboring second carbon atom shows a relatively negative charge (δ^−^) and the next carbon atom shows a positive charge (δ^+^), sequentially. Due to this electrical gradient, it is possible that *H. pylori* urease with its negative charges (such as thiol radicals) might bind to the β-position carbon of zerumbone. Our results show that zerumbone dimerized, trimerized, or tetramerized with both *H. pylori* urease A and urease B molecules, and that this formation of complex inhibited *H. pylori* urease activity. Although zerumbone did not affect either gene transcription or the protein expression of urease A and urease B, our study demonstrated that zerumbone could effectively dimerize with both urease molecules and caused significant functional inhibition of urease activity. In short, our findings suggest that zerumbone may be an effective *H. pylori* urease inhibitor that may be suitable for therapeutic use in humans.

## 1. Introduction

According to the WHO in 2018, gastric cancer is the fifth most common malignancy worldwide and the third leading cause of cancer-related morbidity [1]. Among certain factors responsible for the high gastric cancer rate, *Helicobacter pylori* (*H. pylori*) infection is a common cause and prevalent in East-Asian countries, including South Korea, Japan and China [2]. To eradicate *H. pylori* infection in South Korea, physicians have adopted a standard triple therapy that typically includes one proton-pump inhibitor (PPI) and two antimicrobial agents administered over the course of 10–14 days [3,4]. Unfortunately, antibiotic resistance has become more prevalent over time and has resulted in a failure to eradicate *H. pylori* in South Korea. In particular, clarithromycin resistance has greatly increased in Asia, starting at 15.28% in 2009 and advancing to 32.46% by 2014 [5].

Urease is an enzyme that catalyzes the hydrolysis of urea into carbon dioxide (CO_2_) and ammonia (NH_3_) [6]. *H. pylori* urease exists as a heterodimer of the structural subunits urease A (UreA) and urease B (UreB), which are arranged in a dodecameric ((AB)_3_)_4_ structure with two nickel ions (Ni^2+^) bound by each urease dimer [7]. The catalytic activity of urease only becomes active after nickel is inserted into the active site of the urease B subunit by the action of four accessory proteins (urease D–G) [8,9]. *H. pylori* can survive in hostile pH conditions due to its abundant secretion of urease, and despite not being an acidophile [6], can reach and colonize the gastric mucosa in humans [10].

Zerumbone is a major constituent of the tropical zingiberaceous plant *Zingiber zerumbet* (also known as shampoo ginger) [11]. The rhizomes, which contain large amounts of zerumbone, have been used as an anti-inflammatory folk medicine in Southeast Asia as far back as the early second century [12]. Zerumbone is well-known for its pharmacological activities which include antioxidant [13], anti-inflammatory [14] and anti-cancer effects [15]. Vishwanatha et al. reported the antimicrobial activity of zerumbone against *Staphylococcus epidermidis*, *Escherichia coli*, *Aspergillus oryza,* and *A. niger* [16]. The antibacterial effect of zerumbone against *H. pylori* has been reported by Sidahmed et al., but only the results for the minimal inhibitory concentration (MIC) were studied in any real detail [17]. Herein, we investigated the effects of zerumbone on the protein expression of virulence factors (including CagA) in *H. pylori* via two-dimensional electrophoresis (2-DE) analysis [18]. Additionally, we investigated the effects of zerumbone on urease activity, which is known to be essential for *H. pylori* survival in vitro.

## 2. Results

### 2.1. Confirmation of the Minimal Inhibitory Concentration of Zerumbone

**Figure 1:**
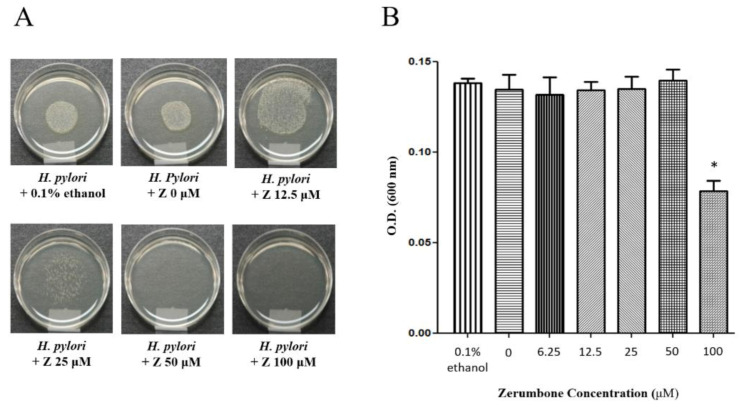
Confirmation of minimal inhibitory concentration of zerumbone. (**A**) *H. pylori* 60190 was inoculated into Mueller–Hinton agar containing 10% bovine serum and zerumbone at various concentrations (6.25, 12.5, 25, 50 and 100 μM). (**B**) *H. pylori* 60190 (1 × 10^8^ CFU/mL) was cultured in Mueller–Hinton broth containing 10% bovine serum and varying concentrations of zerumbone for 3 days (* *p* < 0.05).

*H. pylori* strain 60190 was inoculated into Mueller–Hinton agar containing 10% bovine serum and zerumbone at various concentrations (6.25, 12.5, 25, 50, and 100 μM, Figure 1A). As Figure 1A shows, the minimal inhibitory concentration (MIC) was 50 μM in agar (Figure 1A). *H. pylori* was also cultured in Mueller–Hinton broth containing 10% bovine serum and varying concentrations of zerumbone for 3 days (Figure 1B); the MIC of zerumbone in the broth dilution was 100 μM (* *p* < 0.05, Figure 1B). We found a relatively lower inhibitory concentration in the agar dilution than in the broth dilution method. Therefore, we determined the concentration of zerumbone below the MIC appropriate to measure urease activity through repeated preliminary experiments; 20 μM of condition to the maximum.

### 2.2. The Expression of Urease A and Urease B after Zerumbone Treatment

**Figure 2:**
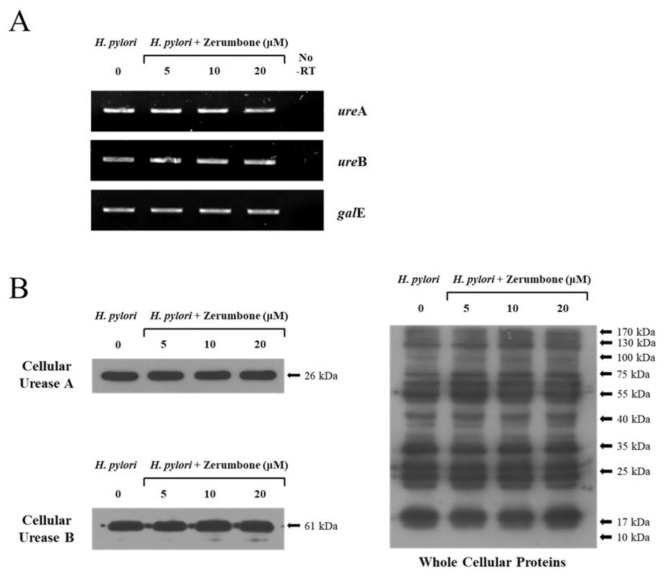
The expressions of urease A and urease B were not affected by zerumbone treatment. (**A**) *H. pylori* was cultured in broth with varying concentrations of zerumbone (0, 5, 10, and 20 μM). RT-PCR was performed and the transcription levels of *ure*A and *ure*B genes were unchanged regardless of treatment. UDP-galactose 4 epimerase gene (*gal*E) was utilized as a control. (**B**) Western blot analysis was performed using anti-*H. pylori* urease antibodies; the expression levels of urease A and urease B were found to be unchanged. To establish a control for comparing the relative change of urease A and urease B with other proteins, we utilized anti-*H. pylori* whole internal protein antibodies.

*H. pylori* was treated with varying concentrations of zerumbone in a broth culture (0, 5, 10, and 20 μM). After three days, RT-PCR was performed for bacterial RNA and the transcription levels of the *ure*A and *ure*B genes were found to be unchanged under all conditions. The UDP-galactose 4 epimerase gene (*gal*E) was utilized as an internal control (Figure 2A). We also performed Western blot analysis using anti-*H. pylori* urease antibodies in order to analyze the expression levels of urease A and urease B; no quantitative changes in the expressions of either protein were detected (Figure 2B). Anti-*H. pylori* whole antibodies were used as an internal control and were designed by our lab as previously published (see Materials and Methods). These whole antibodies were utilized to compare the relative change of urease A and urease B to other proteins (Figure 2B).

### 2.3. Zerumbone Inhibited H. pylori Urease Activity

**Figure 3:**
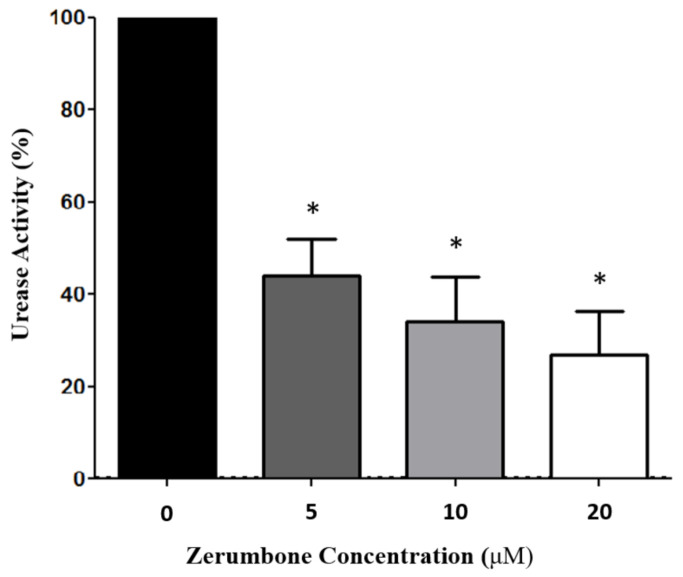
Zerumbone inhibited *H. pylori* urease activity. Urease activity was determined by measuring ammonia production using the indophenol method. *H. pylori* was grown in culture and treated with 0, 5, 10, or 20 μM zerumbone. Urease activity was found to decrease in a dose-dependent manner to 56%, 66%, and 73% of the untreated control, respectively (* *p* < 0.05).

Urease activity was determined by measuring ammonia production using the indophenol method. *H. pylori* was cultured and treated with either 0, 5, 10, or 20 μM zerumbone concentrations. Urease activity was found to decrease in a dose-dependent manner (* *p* < 0.05) to 56%, 66%, and 73% of the untreated control, respectively (Figure 3).

### 2.4. Urease Submolecules (Urease A and Urease B) Formed a Dimer, Trimer, or Tetramer with Zerumbone

**Figure 4:**
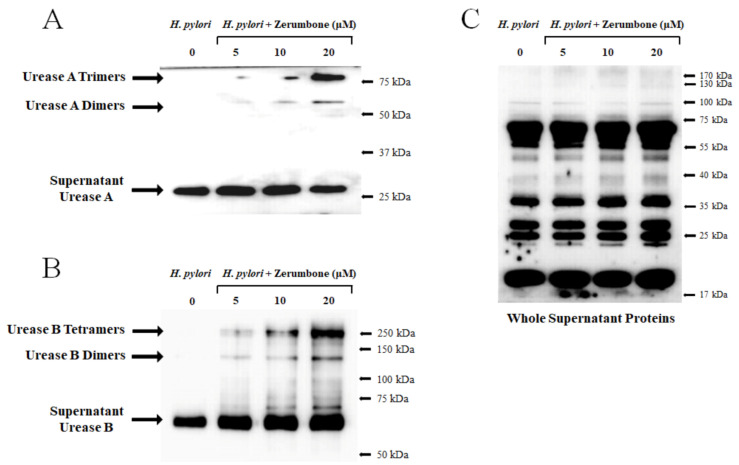
Both urease A and urease B formed dimers, trimers, or tetramers with zerumbone. A native-PAGE assay was performed using culture supernatant proteins. (**A**) The expression of urease A protein (26 kDa) showed almost no change or slightly decreased dose-dependently. On the other hand, anti-urease A antibody-positive proteins were detected around 52 kDa and 78 kDa, which is about two or three times the size of urease A. (**B**) Urease B also showed dimerization, trimerization, or tetramerization with zerumbone. Anti-urease B antibody-positive proteins were detected around 61 kDa, 121 kDa and 244 kDa, which is once again about twice to three times the size of urease B. (**C**) To establish a control for comparing the relative change of urease A and urease B with other proteins, anti-*H. pylori* whole protein antibodies were utilized.

Using the same culture and zerumbone treatment conditions as above, cultured supernatant was collected and concentrated before being utilized for the analysis of secretory urease protein expression. As SDS-free reagents were required to prevent the decomposition of the zerumbone–urease dimers, a native-PAGE process was used for this experiment (Figure 4). The expression of urease A protein (26 kDa) showed almost no change or slightly decreased in a dose-dependent manner (Figure 4A). On the other hand, anti-urease A antibody-positive proteins were detected around 52 and 78 kDa, which is between two and three times the size of urease A (Figure 4A). Urease B also showed dimerization and tetramerization with zerumbone (Figure 4B). Anti-urease B antibody-positive proteins were also detected around 122 and 244 kDa, which is once again about two to four times the size of urease B (Figure 4B).

## 3. Discussion

*H. pylori* produces an abundant amount of urease in order to neutralize the acidic gastric environment via the hydrolysis of urea into ammonia and carbon dioxide [6]. Although it is commonly thought that *H. pylori* favors an acidic environment, it did not exhibit sufficient viability in low pH conditions in our preliminary experiments (Supplementary Data 1). Viable *H. pylori* 60190 cells should secrete urease, but when grown in a pH range between 2~6, *H. pylori* could not survive or produce sufficient urease to increase the environmental pH (Supplementary Data 1). This suggests that *H. pylori* prefers a neutral pH, and thus the function of urease is pivotal for the settlement and growth of *H. pylori*.

The current study has confirmed that zerumbone possesses anti-urease activity. Although both the transcription of *ure*A and *ure*B genes and the expressions of urease A and urease B proteins were not affected by zerumbone treatment (Figure 2), zerumbone showed significant inhibitory effects on *H. pylori* urease activity. Urease activity dropped to 56%, 66%, and 73% of the control in a dose-dependent manner, as shown in Figure 3 (* *p* < 0.05). The structural characteristics of zerumbone are likely responsible for these actions.

**Figure 5:**
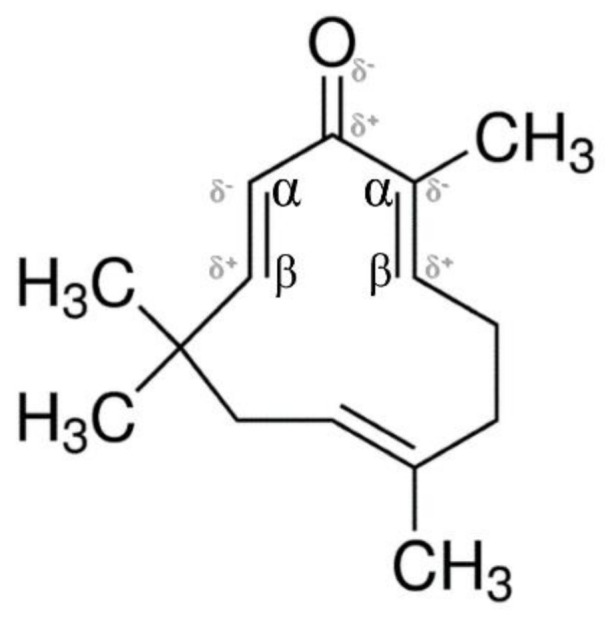
The electrical structure activity relationship of zerumbone. Due to the difference in electronegativity between O and C, the β-position carbon has a positive charge (δ^+^).

**Figure 6:**
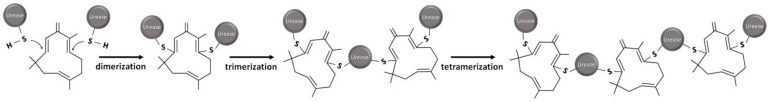
Hypothetical principle for the formation of urease–zerumbone dimer, trimer, and tetramer. Due to the electrical gradient of zerumbone’s structure, it is possible that *H. pylori* urease with its negative charges (such as thiol radicals) might bind to the β-position carbon of zerumbone, sequentially.

In this study, we investigated the ability of a natural compound extracted from *Zingiber zerumbet* Smith, zerumbone to inhibit the urease activity of *H. pylori* by formation of urease-dimers, trimers, or tetramers (Figure 4A,B). As the oxygen atom possesses stronger electronegativity than the first carbon atom bonded to it, in the zerumbone structure, the neighboring second carbon atom at α-position shows a relatively negative charge (δ^−^), and the next carbon atom at β-position shows a positive charge (δ^+^), sequentially (Figure 5). Due to this electrical gradient, it is possible that *H. pylori* urease with its negative charges (such as thiol radicals) might bind to the β-position carbon of zerumbone (Figure 6). Zerumbone exhibits electrical properties based on the above principles and it is believed that it inhibits the activity of urease by forming dimers, trimers, or tetramers with ureases by combining with the -SH radicals present on *H. pylori* urease molecules (Figure 6) [19]. Thus, we thought that the dimerization or more competitive bindings were found in Figure 4A,B.

Our experimental procedures involved numerous trials and errors. The electrical features possessed by zerumbone potentially created the conditions to allow other sulfuric radicals to bind to the β-position carbon. With that in mind, ethanol was used instead of dimethyl sulfoxide (DMSO) when dissolving zerumbone. In addition, since SDS could potentially decompose the zerumbone–urease dimer, reagents containing SDS were excluded from all assays. In our results, we could successfully detect the formation of dimer, trimer, or tetramer through zerumbone with both urease A and urease B. In Figure 4A, urease A-specific antibody bindings of the sizes considered to be 26, 52 and 78 kDa were observed. In the case of urease B, it was possible to confirm the binding of urease B-specific antibodies, which were considered to be 61, 122, and 244 kDa, as shown in Figure 4B. These are evidence of the formation of dimers, trimers, or tetramers by zerumbone.

Urease is one of the primary etiological agents in many Gram-negative bacteria (*Proteus vulgaris*, *Proteus mirabilis*, *Klebsiella pneumoniae*, *Klebsiella oxytoca*) and has also been identified in one Gram-positive bacterium (*Staphylococcus saprophyticus*) [20,21,22]. Over the preceding decades, numerous synthetic compounds have been investigated as potential urease inhibitors; unfortunately, most of these compounds were too unstable or toxic to use in humans [23,24]. Acetohydroxamic acid (AHA) is a well-known urease inhibitor and has been used to treat urinary tract infections, where it works by preventing urine alkalization [25,26]. However, as noted with many of the other previously developed compounds, AHA had severe side effects such as teratogenicity [27] as well as causing psychoneurological and musculo-integumentary symptoms [28,29]. Therefore, the search for novel and useful urease inhibitors continues. In this study, we found that zerumbone effectively inhibited urease activity via dimerization, trimerization, or tetramerization with the *H. pylori* urease subunits. Given this mechanism of action, zerumbone may represent a potentially useful urease inhibitor that does not cause the wide range of side effects seen in previous compounds. Now, we are preparing in vivo application using *H. pylori*-infected Mongolian gerbils.

## 4. Materials and Methods

### 4.1. Bacterial Culture and Determination of MIC

The *H. pylori* reference strain ATCC 49503 was purchased from the American Type Culture Collection (ATCC, Manassas, VA, USA). *H. pylori* was grown on Brucella agar plates (BD Biosciences, Franklin Lakes, NJ, USA) supplemented with 10% bovine serum (BRL Life Technologies, Grand Island, NY, USA) under microaerophilic and 100 percent humidity conditions at 37 °C and inspected after 3 to 5 days. For the agar dilution test, 10 μL of the *H. pylori* suspension (McFarland 2.0) was placed on the Mueller–Hinton agar (BD Bioscience, Franklin Lakes, NJ, USA) supplemented with 10% bovine serum including various concentrations of zerumbone. For the broth dilution test, zerumbone was added with *H. pylori* (McFarland 0.5) in Mueller–Hinton broth (BD Bioscience, Franklin Lakes, NJ, USA) for 72 h before the final optical density (600 nm) of the bacterial suspension was measured via spectrophotometry. In order to determine the MIC, bacteria were exposed to various concentrations of zerumbone (Sigma-Aldrich, St. Louis, MO, USA) in culture with Mueller–Hinton agar and broth (BD Bioscience, Franklin Lakes, NJ, USA) containing 10% bovine serum for 3 days. The minimal inhibitory concentration (MIC) was determined based on the lowest concentration that resulted in growth inhibition. Zerumbone was dissolved in an ethanol solvent, not dimethyl sulfoxide (DMSO), in order to exclude the possibility of zerumbone binding with sulfur (S). For the vehicle control group, equal volumes of ethanol were administrated into cultures.

### 4.2. RNA Extraction and Reverse Transcriptase-Polymerase Chain Reaction (RT-PCR)

*H. pylori* was grown in Muller–Hinton broth with the indicated concentrations of zerumbone (5, 10 and 20 μM) for 72 h. Cultured *H. pylori* were then washed twice with sterile saline and total RNA was extracted using Trizol reagent (Invitrogen, Carlsbad, CA, USA) as described in the manufacturer’s instructions. *Gal*E (UDP-galactose 4-epimerase) was used an internal control. The PCR primer sequences used in this study are as follows: *ure*A-F, 5′-GCCAATGGTAAATTAGTT-3′; *ure*A-R, 5′-CTCCTTAATTGTTTTTAC-3′; *ure*B-F, 5′-TCTATCCCTACCCCACAACC-3′; *ure*B-R, 5′-CCATCCACGAACACATGGTA-3′; *gal*E-F, 5′-ATGGCATTATTATTCACAGG-3′; *gal*E-R, GCTCCATAAGGATTAATGGG-3′.

### 4.3. Protein Extraction and Western Blot

*H. pylori* cultured with zerumbone for 72 h was lysed with radio immunoprecipitation assay (RIPA) lysis buffer (Millipore, Billerica, MA, USA) containing a protease inhibitor cocktail. The cell lysates were then incubated on ice for 10 min, centrifuged at 14,000 rpm at 4 °C for 10 min, and finally the supernatants were collected. Protein samples were separated by sodium dodecyl sulfate (SDS)-polyacrylamide gel electrophoresis and transferred to a nitrocellulose membrane over 90 min at 400 mA. For native-PAGE, a 6.5% (*w/v*) separating gel in 7.5 pH Tris-HCl buffer and a 3% (*w/v*) stacking gel in 5.5 pH Tris-phosphate acid buffer were prepared [30]. The protein sample was dissolved in native sample buffer (62.5 mM Tris-HCl pH 6.8, 10% glycerol, and 0.1% bromophenol blue) without boiling and loaded onto the native-PAGE gel. A total of 0.01 M Tris-Barbital was used as the electrode buffer (pH 7.0) and electrophoresis was conducted at a constant voltage (100 V) at 4 °C until the sample reached the end of gel after 4–5 h. The protein bands were then transferred onto a nitrocellulose membrane for 90 min at 400 mA. Antibodies to detect urease A and urease B were purchased from Santa Cruz Biotechnology (Dallas, TX, USA) and the polyclonal antibody against whole *H. pylori* (ATCC 49503) was produced as previously described [31]. 

### 4.4. Urease Activity Test

Supernatants from the *H. pylori* cultured with zerumbone for 72 h were collected. An amount of 5 μL of 20% urea (Duksan Pure Chemical, South Korea) was added and incubated at 37 °C. After 10 min, the amount of ammonia was measured by an ammonia assay kit (Asan Pharmaceutical, Seoul, South Korea). Briefly, 400 μL of deproteinization solution was added, vortexed, and centrifuged at 2500 rpm for 5 min. An amount of 100 μL of supernatant was mixed with 100 μL of phenol (40 g/L), 50 μL of sodium hydroxide (35.6 g/L) and 100 μL of sodium hypochlorite (10%) and incubated at 37 °C for 20 min. The absorbance was measured at 630 nm wavelength using NanoQuant Infinite M200. 

### 4.5. Statistical Analysis

Data in the bar graphs are presented as mean ± standard error of mean (SEM). All statistical analyses were performed using GraphPad Prism 7.0 software (GraphPad Software, San Diego, CA, USA). All the data were analyzed by unpaired Student’s *t*-test and a *p* < 0.05 was considered to be statistically significant. 

## Data Availability

The data that support the findings of this study are available from the corresponding author upon reasonable request.

## Supplementary Materials

The following are available online. Figure S1: *H. pylori* could not survive and secret sufficient urease in acidic environments (pH 2~6).
