# Supplementary material for: Zerumbone Inhibits Helicobacter pylori Urease Activity
Source: Molecules. 2021 May 1;26(9):2663. doi: 10.3390/molecules26092663 (PMC8124612; doi:10.3390/molecules26092663)
Supplement: Supplementary file 1 [file molecules-26-02663-s001.zip › molecules-1170351-supplementary.pdf]

Figure Legends for Supplementary data

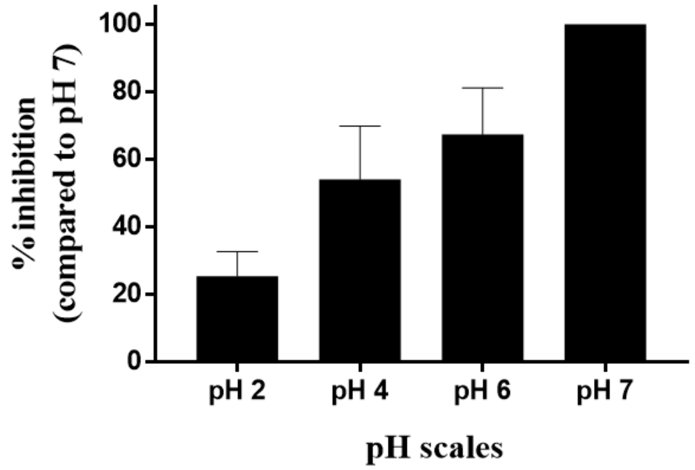

Supplementary data 1. *H. pylori* could not survive and secret sufficient Urease in acidic environments (pH 2~6). *H. pylori* had been pretreated with various scales of pH for 1 hour. Then collected *H. pylori* cells were cultured in preferring media for 6 hours. After the culture, urease test was carried out by adding 2 ml of urease test solution (7 mM phosphate buffer pH 6.8, 110 mM urea, 10 mg/L phenol red). After 1 hour, absorbance values at 540 nm were recorded with a Nano-Quant spectrophotometer. *H. pylori* secreted relatively less ratio of Urease (11, 33, 54%, respectively) when they were grown in pH 2, 4, and 6 range, than when were cultured in pH 7 (neutral).
